# Sample Size Requirements of Glaucoma Clinical Trials When Using Combined Optical Coherence Tomography and Visual Field Endpoints

**DOI:** 10.1038/s41598-019-55345-x

**Published:** 2019-12-11

**Authors:** Zhichao Wu, Felipe A. Medeiros

**Affiliations:** 1 Duke Eye Center and Department of Ophthalmology, Duke University School of Medicine, Durham, North Carolina USA; 2 Department of Ophthalmology, University of California, San Diego, La Jolla, California USA; 3 Centre for Eye Research Australia, Royal Victorian Eye and Ear Hospital, East Melbourne, Australia; 4 Ophthalmology, Department of Surgery, The University of Melbourne, Melbourne, Australia

**Keywords:** Eye diseases, Optic nerve diseases, Outcomes research, Clinical trial design, Translational research

## Abstract

Glaucoma clinical trials using visual field (VF) endpoints currently require large sample sizes because of the slowly-progressive nature of this disease. We sought to examine whether the combined use of VF testing and non-invasive optical coherence tomography (OCT) imaging of the neuroretinal tissue could improve the feasibility of such trials. To examine this, we included 192 eyes of 121 glaucoma participants seen at ≥5 visits over a 2-year period to extract real-world estimates of the rates of change and variability of VF and OCT imaging measurements for computer simulations to obtain sample size estimates. We observed that the combined use of VF and OCT endpoints led to a 31–33% reduction in sample size requirements compared to using VF endpoints alone for various treatment effect sizes. For example, 189 participants would be required per group to detect a 30% treatment effect with 90% power with combined VF and OCT endpoints, whilst 276 and 285 participants would be required when using VF and OCT endpoints alone respectively. The combined use of OCT and VF endpoints thus has the potential to effectively improve the feasibility of future glaucoma clinical trials.

## Introduction

Due to the slowly progressive nature of glaucoma, there have been concerns that short-term clinical trials of new treatments compared to current treatments would have prohibitively large sample size requirements when using visual field endpoints. This is especially the case given the known variability of perimetry^1–4^, where its progressive changes can require many years to detect at the individual level^5^.

Optical coherence tomography (OCT) imaging has emerged in recent years as a powerful technique to monitor progression in glaucoma eyes, through enabling the neuroretinal tissue to be non-invasively visualized and quantified. There has been an accumulating body of evidence that suggests how structural measurements on OCT imaging have been associated with the future development of visual field abnormalities or visual field progression^6–14^. Given its prognostic value and biological plausibility (i.e. providing a direct estimate of retinal ganglion cell loss in glaucoma), neuroretinal measures on OCT imaging show promise as a useful outcome measure that could be used in conjunction with visual field endpoints to improve the feasibility of glaucoma trials^15,16^.

We have also previously observed two methodological aspects that could reduce sample size requirements in glaucoma trials. First, we showed that using a between-group trend-based analysis of the outcome measures instead of the conventional individual-based event-based analysis, resulted in major reductions in the required sample size^17^. Second, we used a testing paradigm that clustered visits at the bookends of the trial period (as originally suggested by Crabb *et al*.^18^), as opposed to evenly-spacing the tests, and these changes in the analytical methods and trial design further reduced sample size requirements^19^.

This study therefore sought to determine whether the combined used of visual field and OCT imaging endpoints could improve the feasibility of future glaucoma clinical trials by reducing the sample size requirements compared to using conventional visual field endpoints alone, when incorporating the two abovementioned methodological aspects.

## Results

### Participant characteristics

A total of 192 eyes from 121 participants with glaucoma were included to obtain estimates of the rates of visual field mean deviation (MD) and global retinal nerve fiber layer (RNFL) thickness change and its measurement variability, and had a mean ± standard deviation (SD) baseline age of 68 ± 11 years (range, 34 to 85 years), and were seen over 5.8 ± 1.5 visits over the 2-year follow-up period. The median (interquartile range [IQR]) baseline MD and pattern standard deviation (PSD) of these eyes was −2.73 decibels (dB; −7.44 to −0.40 dB) and 2.89 dB (1.83 to 8.09 dB) respectively, and their average global RNFL thickness was 75 ± 18 μm (range, 34 to 123 μm). For the eyes included in the simulations (having a baseline MD ≥ −10 dB), their median rate of MD change was −0.09 dB/year (−0.75 to 0.30 dB/year) and mean rate of global RNFL thickness change was −0.5 ± 1.8 μm/year.

### Sample size requirements using different outcome measures

The sample size needed per group to identify different treatment effects using the visual field MD and OCT global RNFL thickness values as the outcome measure in isolation or combined is shown in Table 1. Using both outcome measures together reduced the sample size requirements by 31–33% compared to using visual fields alone, and by 33–35% compared to using OCT alone.

**Table 1:** Sample size requirements for identifying statistically significant treatment effects using visual field (VF) or optical coherence tomography (OCT) in alone or when combined.

New Treatment Effect	Sample Size Required Per Group		
	VF Only	OCT Only	VF and OCT
20%	647	669	449
30%	276	285	189
40%	146	148	98
50%	86	91	59

**Figure 1:**
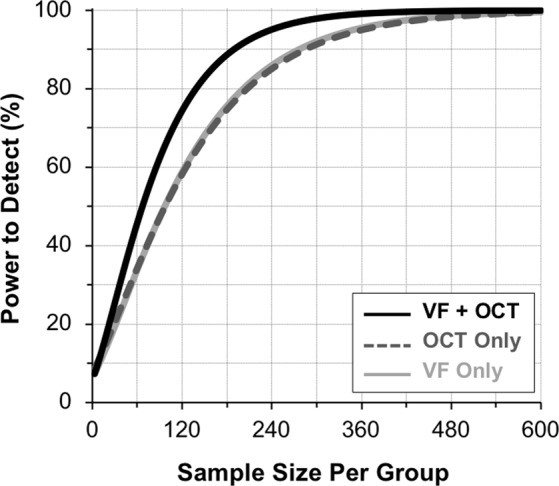
The power to detect a statistically significant beneficial treatment effect plotted against the sample size included per group for a 30% new treatment effect, illustrating how using both the visual field (VF) mean deviation and optical coherence tomography (OCT) global retinal nerve fiber layer thickness measurements as outcome measures (black solid line) performed better than using either the VF (light gray solid line) or OCT (dark gray dashed line) measures alone.

Figure 1 shows the power to detect a statistically significant beneficial treatment effect plotted against sample size per group for the different outcome measures used, for a 30% new treatment effect, illustrating the increased power provided by using both visual field and OCT imaging as outcome measures together.

## Discussion

In this study, we demonstrated that the combined use of visual field and OCT imaging endpoints reduced sample size requirements by 31–33% compared to the conventional use of visual field endpoints alone in a short-term glaucoma clinical trial scenario. These findings underscore the potential value of including OCT imaging as an outcome measure in future glaucoma trials to improve their feasibility.

This is the first study to our knowledge that has evaluated the value of including OCT imaging as an outcome measure in addition to conventional visual field testing. A recent report by Garway-Heath and colleagues^20^ evaluated a method of using OCT imaging data to guide the analysis of visual field progression in glaucoma eyes. When compared to a standard event-based analysis of the visual field data, this method did not reduce the sample size requirements. The authors thus concluded that there was no evidence that OCT imaging and analytical methods used would enable more expedient or smaller trials. The differences in the observations about the potential value of OCT imaging for glaucoma clinical trials may be explained by a few potential factors. Most importantly, the OCT imaging data was used to guide the visual field progression analysis in the recent report^20^, rather than being used independently as an outcome measure. This approach may not have fully capitalized on the information on disease progression provided by OCT imaging. Furthermore, time-domain OCT was used in the previous study^20^ compared to the spectral-domain OCT imaging in this study, and latter has been reported to outperform the former at detecting disease progression in glaucoma eyes^21^.

It should be noted that the value of OCT imaging for reducing the sample size requirements in this study was achieved by using the standard global circumpapillary RNFL thickness measure. However, recent studies have revealed how neuroretinal parameters in the macula and posterior pole (outside of the peripapillary region) could also provide important information about disease progression^10–14,22,23^ that could be exploited to further improve the power to detect a significant treatment effect. Furthermore, analytical methods that limit the evaluation of progression within regions of established glaucomatous damage (to minimize the contribution of measurement variability from non-damaged regions) could further reduce sample size requirements^23–26^.

 It is also important to acknowledge the potential limitations of using OCT imaging as an outcome measure in glaucoma trials; these have been discussed in detail elsewhere^15,16^. Whilst neuroretinal measures show strong biological plausibility for representing the retinal ganglion cells that are lost in glaucoma, they do not directly reflect “*how a patients feels, functions or survives*”^27^, being the definition of a clinical endpoint. Given that neuroretinal measures have been shown to be an important predictor of relevant functional outcomes in glaucoma, they could reasonably be considered as a surrogate endpoint. However, validation of surrogate endpoints is a complex issue and a significant effect in predicting the clinically relevant outcome is only part of the requirement of valid surrogates^28^. In addition, the effect of a proposed treatment on the surrogate must capture a sufficiently large proportion of the effect of the treatment on the clinically relevant endpoint^29,30^. Nonetheless, there is only currently some evidence about the validity of neuroretinal measures as a surrogate endpoint in intraocular pressure lowering treatments in glaucoma^31^, but this evidence in neuroprotective therapies is still not available yet. This is crucial to consider, as it is possible for a novel neuroprotective therapy to preserve neuroretinal tissue without showing a meaningful effect on preserving relevant functional outcomes^15,16^. The interpretation of the efficacy of a potential treatments based on combined OCT and visual field endpoints therefore requires recognition of this important limitation.

It is interesting to observe that sample size requirements for visual fields used in isolation were very similar to those when OCT was used in isolation. This is a surprising finding that seems to go against the common notion that objective testing by OCT would capture disease progression much better than subjective visual field testing. For instance, a previous study suggested that OCT RNFL thickness measurements had a longitudinal signal-to-noise ratio (a normalized measure to enable fair comparisons between different techniques) nearly twice that of visual field MD^32^. This difference may be attributed to the differences in the rate of change of these parameters between different populations. Regardless of this finding, our results show that the combined use of OCT and visual field results as endpoints significantly reduced sample size requirements.

These findings build on our previous body of work that demonstrated that the feasibility of future glaucoma clinical trials could be improved by using different analytical methods^17^ and testing paradigms^19^. It is important to note when interpreting the results of this study that our primary aim was to report the relative value provided by including OCT imaging as an outcome measure in addition to visual field tests, rather than the provision of the actual estimates itself. This is because the actual sample size estimates will vary based on the characteristics of the participants included and the design of the clinical trial (such as the number of tests included, and the testing paradigm used, or whether to include both eyes), or the methods used to obtain the outcome measures (such as the scanning protocol used for OCT imaging).

In conclusion, this study revealed that the combined use of visual field and OCT imaging endpoints reduced the sample size requirements by approximately a third when compared to using visual field endpoints alone. These findings demonstrate how OCT imaging has the potential to markedly improve the feasibility of future glaucoma clinical trials when used as an additional outcome measure.

## Methods

### Participants

The participants included in this study were evaluated in a longitudinal study that evaluated structural and functional changes in glaucoma. Institutional review board approval by the University of California, San Diego was obtained for this study, and it adhered with the Declaration of Helsinki and Health Insurance Portability and Accountability Act. All participants also provided written informed consent after receiving an explanation of the test procedures.

All participants in this study underwent a comprehensive ophthalmologic examination at each visit, including a medical history review, visual acuity measurements, visual field testing, OCT imaging, optic disc stereophotography, slit-lamp biomicroscopy, ophthalmoscopic examination, intraocular pressure measurements and gonioscopy. Participants were all required to have a best-corrected visual acuity of 20/40 or better, open angles on gonioscopy and be 18 years of age or older. This study only included eyes with glaucoma, defined on the basis of masked grading of the optic nerve appearance on optic disc stereophotographs as described previously^33^. Eyes were also considered to have glaucoma if there was evidence of progressive change on masked grading of the optic disc stereophotographs^34^, or if they had a history of three or more consecutive abnormal visual field tests (defined as having a glaucoma hemifield test being outside normal limits or a pattern standard deviation value at *P* < 0.05)^35^. Participants with any other ocular or systemic disease other than glaucoma that could affect the visual field and OCT imaging results were excluded from this study. This study also included only eyes with at least five reliable and good quality visual field tests and OCT imaging results (as described further below) over a two-year follow-up period.

### Visual field testing

All visual field tests were performed using the 24–2 strategy with the Swedish Interactive Thresholding Algorithm, Standard on the Humphrey Field Analyzer II-i (Carl Zeiss Meditec, Inc., Dublin, CA, USA). The results were evaluated for the presence of testing artifacts, including fatigue or learning effects, inattention, improper fixation or rim or eyelid artifacts, and were excluded if such artifacts were present. Glaucoma eyes with visual field results that were suggestive of another disease (e.g. homonymous hemianopia) were also excluded from this study. Any visual field test with >33% fixation losses or false negative errors (except for false negative errors when visual field MD was < −12 dB), or with >15% false positive errors were considered unreliable and also excluded.

### Optical coherence tomography imaging

OCT imaging was performed using the Spectralis HRA + OCT (Heidelberg Engineering GmbH, Heidelberg, Germany) device, with a circle scan having a diameter of 12° centered on the optic disc obtained from all eyes. These scans contained 1536 A-scans (high-resolution mode), and were acquired using the Automatic Real-time Tracking mode, averaging at least nine scans at the same location. Each scan during the follow-up visits from baseline were taken at the same retinal location using the image registration and eye tracking software of the device. Only scans with a quality score ≥ 15 and without imaging artifacts (e.g. clipping) were included.

To account for the normal age-related changes when evaluating progression on OCT imaging^36^ (described further below), data from 519 tests from 200 eyes of 114 healthy participants who underwent the same OCT imaging were evaluated. These participants were on average 62.7 ± 14.1 years old (range, 21 to 96 years old), and the mean global RNFL thickness change over time was −0.27 μm/year (95% confidence interval = −0.36 to −0.17 μm/year) for these healthy eyes, estimated using a linear mixed model.

### Data analysis

#### Overview

To simulate progression scenarios necessary for sample size calculations, this study used longitudinal visual field and OCT imaging data from glaucoma eyes under routine clinical care to obtain estimates of their rates of change and measurement variability. The sample size estimates were then obtained by comparing two groups in a simulated clinical trial using linear mixed models (LMMs), which we have demonstrated recently to substantially reduce sample size requirements compared to survival analysis of endpoints based on event-based analysis (such as the Guided Progression Analysis [GPA]; Carl Zeiss Meditec, Inc., Dublin, CA, USA)^17^.

#### Computer simulations

**Figure 2:**
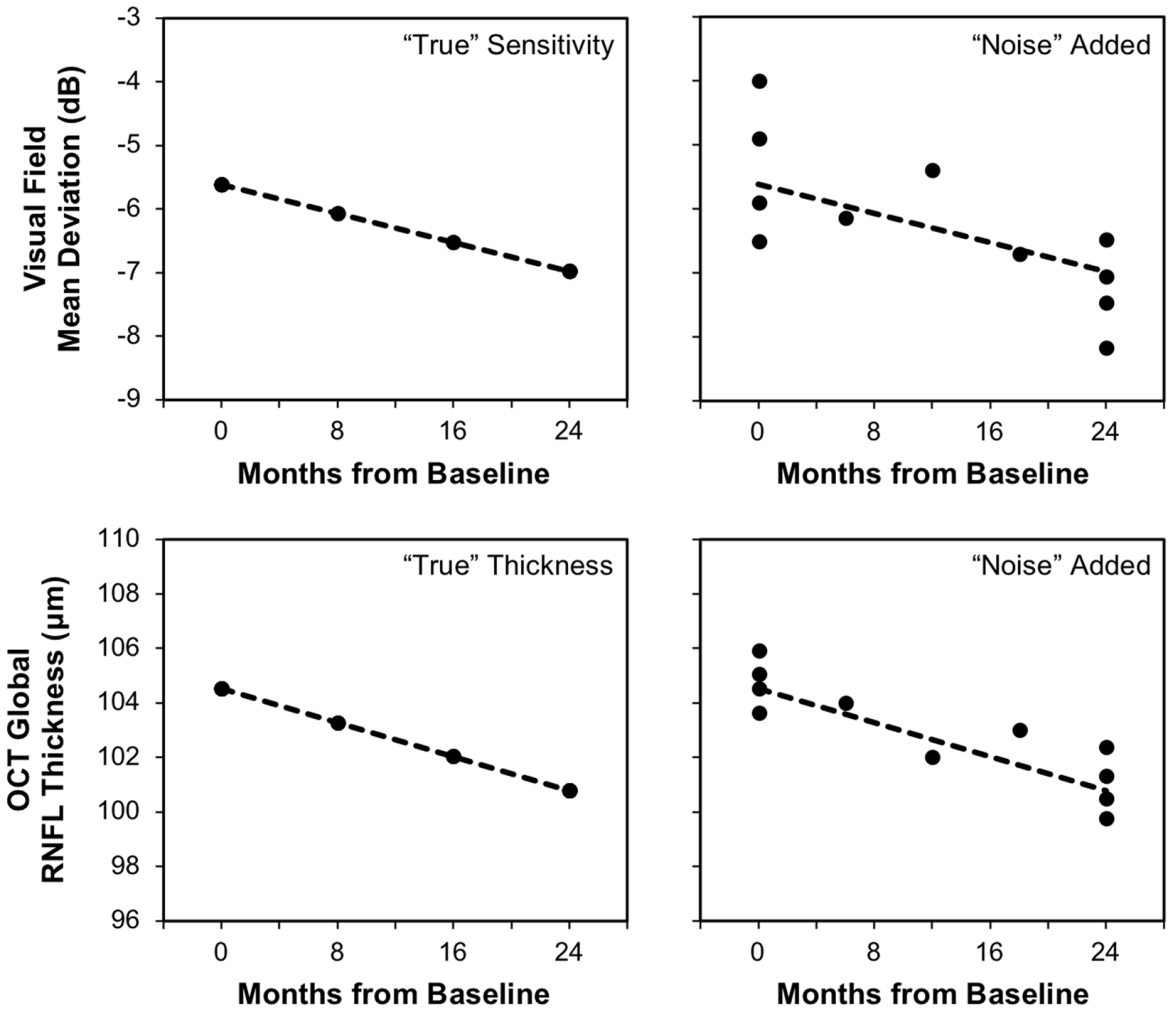
Illustration of the method used to simulate visual field mean deviation (top) and optical coherence tomography (OCT) global retinal nerve fiber layer (RNFL) thickness (bottom) values. For each example, the “true” values were estimated at each time point based on the slope and intercept estimates from linear regression analysis of an eye in the clinical cohort. Measurement variability (or the “noise” component) was then randomly added to each of these “true” values.

To reconstruct “real-world” visual field and OCT imaging results, a computer simulation model was developed in a similar manner as described recently^5^. Briefly, estimates of the baseline and rates of visual field MD and global RNFL thickness change over time (the “signal” component) were obtained from the intercept and slopes of ordinary least squares linear regression models fitted to these values over time respectively for each eye. Note that the baseline and rates of visual field MD and OCT global RNFL thickness change over time obtained from each eye were always used together in the simulations described further below to account for their correlations within the same eye of a participant. Estimates of measurement variability (the “noise” component) were then obtained from the residuals of these linear regressions, by subtracting the measured value from the fitted values. The residuals were then grouped into 1-dB bins for visual field MD and 10-μm bins for global RNFL thickness, based on their respective fitted values. These components were then used to reconstruct “real-world” results by first calculating the “true” visual field MD and global RNFL thickness at each time point by using their estimated slopes and intercepts of each eye. Measurement error was then introduced by randomly selecting a residual based on the “true” value to be added at each time point. The simulations were only performed using eyes with a baseline MD ≥ −10 dB, to recreate a clinical trial scenario in eyes with the early to moderate stages of glaucoma. This cut-off was also chosen since global RNFL thickness values tend to become asymptotic (i.e. reach a floor effect) for MD values approximately below −10 dB^37^, and this outcome measure would thus be less effective for eyes with this severity of glaucoma. An example of this simulation for both visual field MD and global RNFL thickness is shown in Fig. 2.

#### Clinical trial scenario

The testing protocol used in these simulations is based on a novel clustered paradigm that we described in our recent study^19^ and a previous study^18^. However, the testing protocol used in this study was modified to include only 11 tests over a 2-year follow-up period, including 4 tests each at baseline and trial end, and 1 test each at the 6-, 12- and 18-month follow-up time points. The cluster of tests at the bookends of the trial period maximizes the power to detect change^18^, whilst the intermediary visits ensure that there is sufficient opportunity to detect progression throughout the trial period. Progression was evaluated at each follow-up time point for visual fields and OCT imaging using ordinary least squares linear regression models. Progression was considered to have occurred on visual field testing if a statistically significant (*P* < 0.05) negative slope for MD values was detected at two consecutive visits. For OCT imaging, progression was considered to have occurred if a statistically negative slope for global RNFL thickness values relative to the mean rate of age-related change was also detected at two consecutive visits. Eyes meeting this progression criterion were excluded for the remainder of a simulated trial, since such eyes typically require a change in their clinical management in a real-world clinical trial scenario.

This study then simulated visual field and OCT imaging results in a clinical trial scenario where a new treatment was added to one of two groups that were both under routine clinical care with currently available therapies. Sample size requirements were then determined for a new treatment that slowed the rate of visual field MD and OCT global RNFL thickness change over time by 20%, 30%, 40% and 50%. Sample size estimates were obtained by simulating 1000 sequences of clinical trials that included between 10 to 1000 participants per group. For each simulated trial, eyes with different baseline and rates of change of visual field MD and global RNFL thickness were randomly selected without replacement from the clinical cohort. The same eyes were included in the two treatment arms in order to match the characteristics of the two groups.

#### Analysis of outcome measures

During each simulated clinical trial, the difference in the rate of visual field MD or global RNFL thickness change over time between the treatment groups were evaluated using LMMs, specifying random intercepts and random slopes to account for the individual-specific deviations from the population-average change. The statistical significance of this difference was determined by evaluating the interaction between the treatment group variable and time, which represents the difference in the rate of change between the two groups. A significant beneficial treatment effect was considered to be present evaluated using visual fields or OCT imaging alone if the average rate of change of the new treatment group was more positive (or slower) compared to the control group, at a two-tailed significance level of *P* < 0.05. In a clinical trial scenario where both measures are used, a Hochberg procedure was used account for false discovery rates with multiple testing^38^. Since only two measures were evaluated in this study, a significant beneficial treatment effect was considered present if the highest *P*-value or second highest *P*-value associated with a reduction in the rate of change in the new treatment group was less than 0.05 and 0.025 respectively.

## Data Availability

The datasets generated during and/or analysed during the current study are available from the corresponding author on reasonable request.
